# Effect of pH-shifting on the Physicochemical Properties of Pea Proteins and Its Effect on the Texture of Hybrid Gels Formed with Casein Micelles

**DOI:** 10.3390/foods14162887

**Published:** 2025-08-20

**Authors:** Raiane Rodrigues da Silva, Luis Henrique de Paula Souza, Lucas Silva de Sousa, Laura Destro Rodrigues, Gustavo Schäfer Nogueira, Luis Gustavo Lima Nascimento, Antônio Fernandes Carvalho

**Affiliations:** InovaLeite Research Group, Department of Food Technology, Federal University of Viçosa (UFV), Viçosa 36570-900, Minas Gerais, Brazil; raiane.r.silva@ufv.br (R.R.d.S.); lucas.sousa1@ufv.br (L.S.d.S.); luisgusta.ln@gmail.com (L.G.L.N.)

**Keywords:** pea protein, casein, pH-shifting, structure, sustainability

## Abstract

Hybrid systems combining animal and plant proteins are promising for developing sustainable, high-protein foods. However, structural incompatibility between proteins like casein and pea protein hinders the formation of stable systems such as gels. This study explores pH-shifting (alkalization at pH 12 followed by neutralization) as an innovative strategy to improve pea protein functionality and compatibility in hybrid gels. Modified pea protein showed increased solubility, reduced particle size, higher zeta potential, and decreased intrinsic fluorescence intensity, indicating conformational changes and exposure of buried tryptophan residues. These structural changes influenced gel behavior depending on the protein ratio (casein/pea—80:20, 50:50, 20:80). Gels with higher pea content showed increased hardness and water-holding capacity, while in casein-rich gels, hardness decreased, likely due to altered protein–protein interactions. This is the first study to systematically apply pH-shifting to enhance the compatibility between pea protein and casein in high-protein gels, integrating structural and functional analyses. The results demonstrate the potential of pH-shifting as a sustainable and effective approach for improving plant protein performance in hybrid formulations.

## 1. Introduction

Climate change has become a growing global concern, with food production being a significant contributor to this issue. It has a considerable impact on greenhouse gas (GHG) emissions, freshwater scarcity, eutrophication, land degradation, and biodiversity loss [1]. In the United Nations 2030 Sustainable Development Goals, the achievement of certain targets, such as those related to Climate Action and Life on Land, highly depends on consumption patterns [1,2]. Furthermore, population growth and concerns about food security, particularly regarding protein production, increase the need for changes in eating habits [3].

Changing a society’s eating habits is not an easy task. Generally, a gradual transition is necessary. As a result, industries and research groups have increasingly focused on hybrid systems as a strategy to introduce new dietary practices [4,5]. For example, several studies have investigated the replacement of animal proteins with plant-based proteins in the food system [6,7,8,9], since animal production requires more land and water, emits more greenhouse gases, and has a lower conversion rate into dietary protein [3].

Among plant-based proteins, pea protein has gained significant attention due to its high yield, low cost, and excellent amino acid profile, particularly because of its lysine and tyrosine content [10], making it a promising candidate for replacing animal proteins. However, combining proteins from different sources, such as milk and pea proteins, can drastically alter the properties of colloidal systems [4,5]. When pea and milk proteins, such as casein, coexist in the same system, their thermodynamic incompatibility induces a competitive dynamic that impacts the structure of colloidal systems, such as in gel formation [11,12]. To enhance the interactions between these two proteins and reduce their incompatibility, several techniques have been employed, including thermal treatment [12,13], ultrasound [14], and high hydrostatic pressure [15]. A promising new approach to further improve their interaction could involve structural modifications induced by pH-shifting.

The pH-shifting technique consists of adjusting the pH to extreme acidic or basic conditions and subsequently returning it to the neutral pH. This process induces the formation of “molten globules”, which are intermediate conformational states during protein unfolding that retain the secondary structure of the native state [16]. In recent years, the effects of pH-shifting on plant proteins have been investigated. Comparing the effect of pH-shifting on pea protein hydrogels formed by heating, Zhu et al. [16] found that shifting the pH to 12 altered the gel microstructure, resulting in a uniform, polymer-like network with higher water-holding capacity (WHC), depending on the treatment duration. Li et al. [17], studying pH-shifting treatments at different pH levels (2, 4, 10, and 12) in peanut protein isolate heat-induced gels, observed that the modification at pH 10 reduced particle size and increased solubility, free sulfhydryl group content, and surface hydrophobicity, making it an effective method for forming gels with different structural properties. In systems based on soy/potato [17], mung bean protein [18], and soy protein [19], pH-shifting, alone or combined with other methods, has also proven effective in modifying protein structures and promoting gel formation. Despite its promise, the effect of pH-shifting on pea protein/casein micelle systems has never been evaluated.

Therefore, this study aims to evaluate the effectiveness of pH-shifting treatment on pea protein and its impact on hybrid gel systems formed by pea protein and casein at different protein ratios, using acid-induced gelation. The experimental approach involves first modifying pea protein using pH-shifting (pH 12) and subsequently mixing it with casein in different ratios (80:20, 50:50, and 20:80 (casein/pea protein)). The effects of this modification are assessed in both the protein suspensions and the gel structures formed by acid gelation, and to the best of our knowledge, this is the first time that pH-shifting has been applied in such a system.

## 2. Materials and Methods

### 2.1. Materials

Pea proteins (Nutralys, F85F, 83%) were kindly donated by Roquette (Lestrem, France), and micellar casein isolate (Lacprodan Micelpure 86.5%) was donated by Arla Food Ingredients (Århus, Denmark). The protein content was determined by the Kjeldahl method [20], with nitrogen conversion factors (N) of 6.25 and 6.38 for pea protein and casein, respectively. 

### 2.2. Methods

#### 2.2.1. Suspension Preparation

The protein suspension was prepared by diluting the protein isolates in deionized water to a concentration of 12% (*w*/*w*). This concentration was chosen to simulate a high-protein yogurt. The suspensions were stirred for at least 12 h in a mechanical agitator to guarantee complete protein hydration. Sodium azide (0.003%) was added to the suspensions to avoid microbiological growth.

#### 2.2.2. pH Modification

The pea protein suspension was modified by adjusting the pH to 12 using 3 M NaOH and maintaining it under agitation for 24 h. After this period, the pH was readjusted to 7 using 3 M HCl [21]. The treatment time was determined through preliminary testing, taking into account the protein solubility after pH adjustment.

Following the modification, the pea protein suspension (PPS) was mixed with the casein micelle suspension (CM) in different protein ratios, as shown in Table 1. The mixtures were stirred for 30 min before analysis. Systems without pH modification were also evaluated.

#### 2.2.3. Solubility

The solubility test was performed according to the method of Li et al. [22], with minor modifications. The suspensions were centrifuged at 3600× *g* for 15 min, and the protein content of the supernatant was determined using the biuret method. The total protein content was also measured before centrifugation. Solubility was then calculated using Equation (1). Bovine serum albumin (BSA) was used as the standard protein.

(1) $$Solubility\;\left(\%\right)=\frac{Protein\;content\;in\;supernatant}{Protein\;content\;before\;centrifugation}\times 100\%$$

#### 2.2.4. Particle Size and Zeta Potential

The particle size distribution and zeta potential were measured with the Zetasizer Nano ZS (Malvern Instrument Ltd., Malvern, UK) according to the method of Li et al. [22]. First, the suspensions were diluted 100 times, and then 1 mL of the sample was injected into the capillary cells. After, the zeta potential and particle size distribution were tested. All measurements were conducted at 25 °C in three independent tests.

#### 2.2.5. Polyacrylamide Gel Electrophoresis

The protein profile was determined by the electrophoresis technique, following the methodology described by Beghdadi et al. [23]. The samples were prepared by a first dilution to 10 mg/mL in deionized water and were then added to the buffer for native conditions (Tris-HCl 0.5M pH 6.8, pH 6.8, glycerol, Bromophenol Blue) and reduced conditions (Tris-HCl 0.5M pH 6.8, SDS, glycerol, β-mercaptoethanol, Bromophenol Blue). The gels (7 × 10) were composed of 4% stacking and 15% separating gels. After gel solidification, 10 µL of the sample was placed in the well, and the gel was run using a running buffer (0.025 M Tris, 0.192 M glycine, 0.1% SDS) at pH 8.3, applying 150 V for 1 h. After the protein migration, gel staining was performed by immersion in a solution of 0.15% Coomassie^®^ Brilliant Blue R-250 dissolved in acetic acid, methanol, and water for 30 min under agitation, followed by discoloration in acetic acid solution (10%).

#### 2.2.6. Intrinsic Fluorescence

The intrinsic fluorescence of tryptophan was analyzed according to the method described by Nascimento et al. [14] with minor modifications. Samples were diluted in deionized water to a final concentration of 10 mg/mL and transferred to a 96-well microplate. The excitation wavelength was set at 280 nm, and the emission spectra were recorded from 280 to 500 nm.

#### 2.2.7. Gel Preparation

The gel preparation was performed according to the method described by Nascimento et al. [12]. To promote the gelation, glucono-delta-lactone (GDL) was added to the suspension and stirred for one minute to ensure complete solubilization. Ultimately, the suspension was incubated in a water bath at 30 °C for 4.5 h, allowing the pH to gradually decrease to 4.5.

#### 2.2.8. Water-Holding Capacity (WHC)

The water-holding capacity was measured according to Nascimento et al. [12], with minor modifications. First, 10 g of gel was prepared in a centrifuge tube at 30 °C. After the formation, the tube was centrifuged at 3600× *g* for 15 min, and the supernatant was carefully removed and weighed. The WHC was calculated by Equation (2).

(2) $$WHC\;\left(\%\right)=\frac{mg-ms}{mg}$$

where mg is the gel mass before centrifugation, and ms is the mass of the supernatant.

#### 2.2.9. Texture Analysis

The gel textural characteristics were analyzed according to Batista et al. [24] using the universal machine test (Instron Corporation, Norwood, MA, USA), after 1 day of storage at 4 °C. A cylindrical probe with 12 mm diameter was displaced perpendicularly under the gel, with a 250 N load cell, compression distance of 60% of the initial height, test speed of 1.00 mm/s, with two penetration cycles, and three repetitions. The hardness, gumminess, and springiness were evaluated.

### 2.3. Statistical Analysis

To confirm the impact of pH-shifting on the ratios, the data were analyzed using analysis of variance (ANOVA) with Statistica 12.0 software (StatSoft Inc., Maisons-Alfort, France). The data were further examined using Tukey’s HSD test at the 5% confidence level to distinguish between means when a significant difference (*p* < 0.05) was seen. Every experiment was carried out at least three times on its own.

## 3. Results and Discussion

### 3.1. Solubility

**Figure 1:**
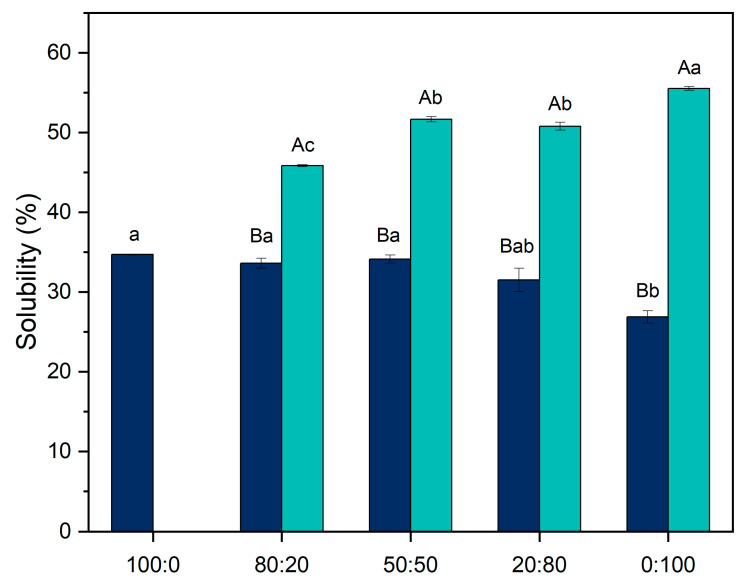
Protein solubility before and after pH-shifting for the ratios 100:0. 80:20, 50:50, 20:80, and 0:100 (casein/pea protein). Dark blue represents the suspensions before the modification, and light blue after the modification. Uppercase letters indicate differences between the treatments in the same ratios, while lowercase letters indicate differences between the ratios under the same treatments. Statistical differences were determined using one-way ANOVA followed by Tukey’s post hoc test (*p* < 0.05).

Protein solubility (Figure 1) is an important parameter for characterizing proteins, as it provides an indication of their techno-functional properties, such as gelation [23]. In the untreated samples, solubility decreased as the proportion of pea protein increased relative to casein (*p*-value < 0.05). A similar trend was observed by Nascimento et al. [11] in which casein micelles exhibited a solubility of 64.0%, while pea protein showed 42.8%. Most of the plant proteins present low solubility comparing to animal proteins, representing a challenge for their application, reinforcing the need for treatments such as pH-shifting to improve their techno-functional properties and enhance their potential in food formulations.

Therefore, after applying the pH-shifting treatment, all ratios showed a significant increase in protein solubility (*p*-value < 0.05). Among them, the 0:100 ratio exhibited the greatest increase, rising from 26.89% to 55.54%. A similar result was reported by Jiang et al. [25], who observed a solubility of 54.94% for pea protein suspension after pH-shifting to pH 12. This similarity demonstrates that pH-shifting has a substantial impact on pea protein solubility and can be effectively used to improve its techno-functional properties. When proteins are exposed to extreme conditions, such as alkaline pH, their normally folded structures unfold, increasing electrostatic repulsion within the system. Upon returning to neutral pH (pH 7), proteins tend to refold. However, some of the original interactions are disrupted, resulting in a “molten globule” state, an intermediate conformation characterized by a preserved secondary structure but a disrupted tertiary structure [15,16,23].

The least affected ratio was 80:20, which showed a solubility of 45.84% after treatment. This smaller increase is likely due to the low proportion of modified pea protein in the system, indicating that casein micelles contribute more significantly to solubility than pea protein in these mixtures.

Besides the modifications caused by pH-shifting, some protein subunits may dissociate from the protein aggregates, contributing to the increase in solubility [24]. Therefore, this contribution was evaluated through particle size analysis.

### 3.2. Particle Size and Zeta Potential

**Figure 2:**
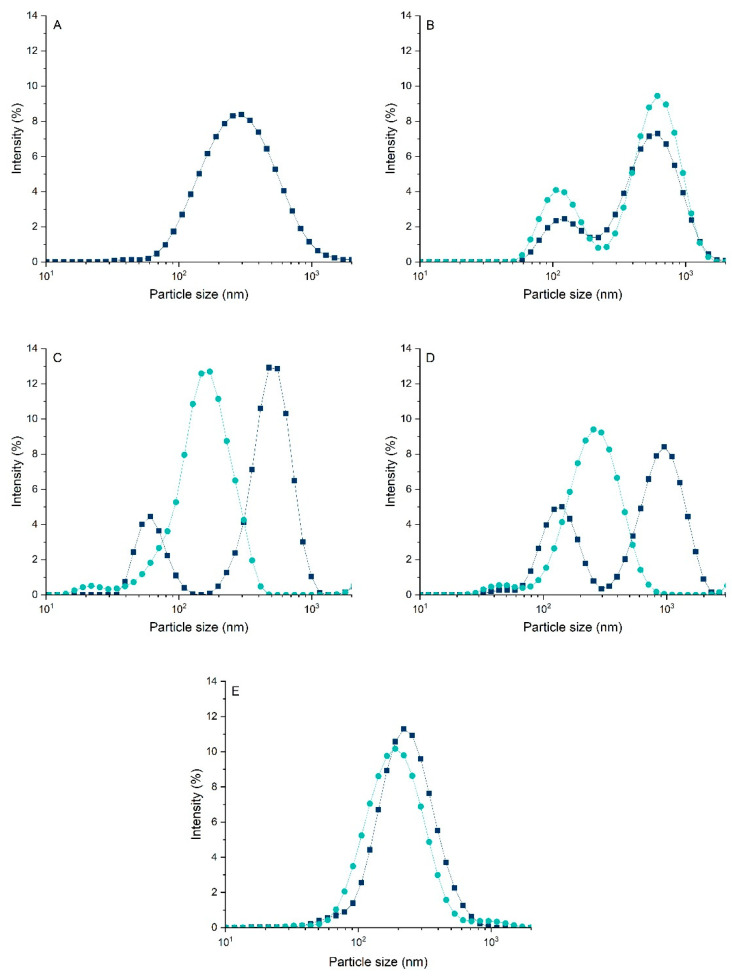
Particle size distribution of protein suspensions: (**A**) 100:0 (**B**) 0:100 (**C**) 20:80 (**D**) 50:50 (**E**) 80:20 (casein/pea protein). Dark blue represents the suspensions before the modification, and light blue after the modification.

Particle size is an important parameter for understanding the effect of a treatment on the techno-functional properties of proteins. One characteristic influenced by changes in particle size is solubility, since smaller protein aggregates have a larger surface area, allowing greater interaction with water, which increases solubility and subsequently impacts techno-functional properties [23]. Therefore, the particle size of the protein suspensions was evaluated, and the results are shown in Figure 2.

Before the treatment, the 0:100, 50:50, and 20:80 ratios exhibited a bimodal distribution. In the 0:100 ratio, the first peak was around 122.4 nm and the second around 615.1 nm. For the 50:50 ratio, the first peak remained at a similar size but with increased intensity, while the second peak was at 955.4 nm. In the 20:80 ratio, the first peak was near 61.2 nm and the second near 553.2 nm. This two-peak distribution is typical of pea protein powder due to the presence of aggregates, which often form during the harsh processing conditions involved in pea flour production [22].

After the treatment, particle size decreased in the hybrid systems (80:20, 50:50, and 20:80). Furthermore, the 50:50 and 20:80 ratios, which initially showed two peaks, presented only a single peak after treatment, indicating a reduction in particle size. This decrease is likely due to dissociation of protein aggregates, resulting in smaller particles and a changed distribution profile [23]. The 80:20 ratio also showed a reduction in particle size. In contrast, no significant change was observed in the 0:100 ratio, suggesting that the improvement in solubility was not solely caused by dissociation of protein aggregates, but also by conformational changes, as supported by the zeta potential results.

**Figure 3:**
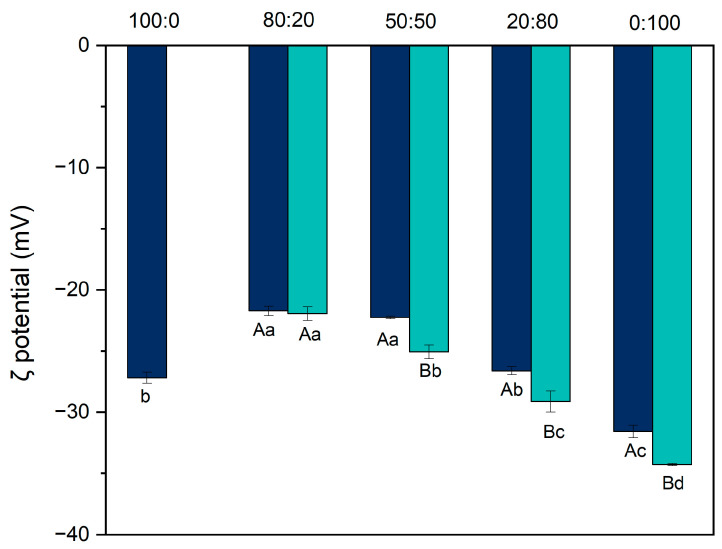
Zeta potential (mV) of protein suspensions for the ratios 100:0. 80:20, 50:50, 20:80, and 0:100 (casein/pea protein). Dark blue represents the suspensions before the modification, and light blue after the modification. Uppercase letters indicate differences between the treatments in the same ratios, while lowercase letters indicate differences between the ratios under the same treatments. Statistical differences were determined using one-way ANOVA followed by Tukey’s post hoc test (*p* < 0.05).

To verify the effect of pH-shifting on protein structure, zeta potential was evaluated. Zeta potential (Figure 3) is an indicator of the electrostatic stability of suspensions, as it quantifies the net surface charge of the particles [13]. All suspensions showed a negative charge, indicating that the surface of the particles contained more negatively charged amino acids than positively charged ones [13,17].

Before pH-shifting, a significant difference was observed among the samples (*p* < 0.01). The hybrid systems at ratios of 80:20, 50:50, and 20:80 exhibited the highest zeta potential values: −21.71 mV, −22.25 mV, and −26.62 mV, respectively. These were followed by the 100:0 ratio (−27.19 mV), while the lowest value was recorded for the pure pea protein suspension (0:100) at −31.58 mV. This pattern is consistent with previously reported results [13,25].

After pH-shifting, the zeta potential increased for most suspensions, except for the 80:20 ratio, where the small amount of modified pea protein was likely insufficient to significantly alter the surface charge. As the proportion of pea protein increased, the zeta potential also increased, reaching −34.09 ± 0.09 mV for the 0:100 ratio. This suggests that the improvement in solubility results from structural changes. Upon returning the system to pH 7, the molten globule-like structure may prevent complete refolding, leaving previously buried or stabilizing groups exposed and negatively charged, thereby increasing the zeta potential [17]. These changes were statistically significant (*p* < 0.01). This interpretation is further supported by the intrinsic fluorescence analysis presented later in this study.

Particle charge is also closely linked to solubility. According to Li et al. [16], as the absolute value of the zeta potential increases, repulsive interactions between molecules also increase, leading to greater system stability by reducing aggregation and ultimately enhancing solubility. When the zeta potential is greater than or equal to ±30 mV, the system tends to be more stable, and particles are less likely to aggregate.

### 3.3. Electrophoresis

**Figure 4:**
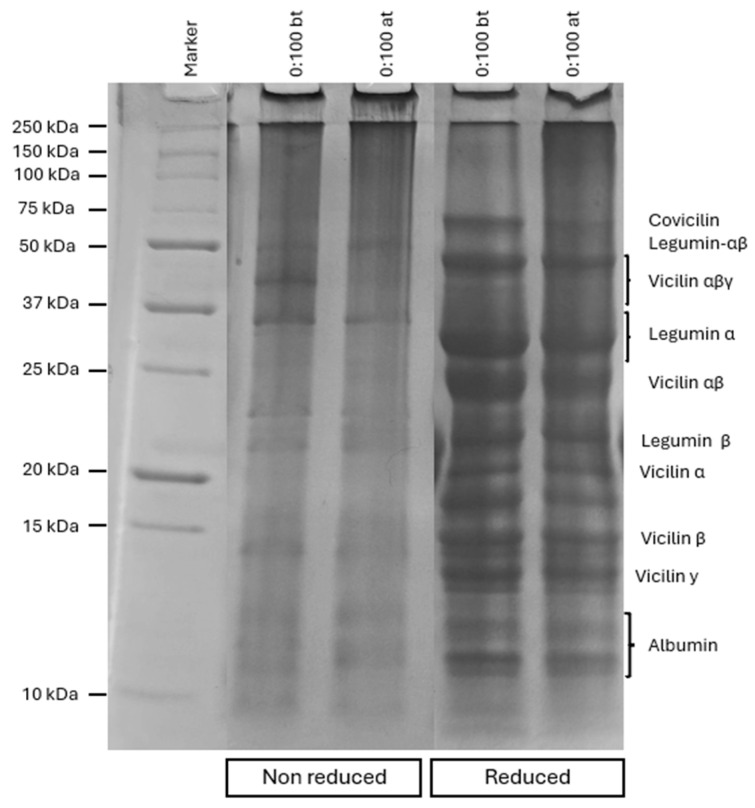
SDS-PAGE gel profiles of pea protein (0:100) under non-reducing and reducing conditions, before (Bt) and after (At) pH-shifting.

The effect of pH-shifting on pea protein subunits can be observed through the electrophoresis profile (Figure 4). Pea proteins are primarily composed of two fractions, albumins (15–25%) and globulins (49–70%), with globulins being the most abundant. Within this fraction, globulins can be further classified based on their sedimentation coefficients into 11S (legumin) and 7S (vicilin) subunits [26].

Pea legumin (11S) is a hexameric globular protein with a molecular mass ranging from approximately 310 to 400 kDa. Each legumin subunit has a molecular mass of ~65 kDa and consists of two polypeptide chains, an acidic α-chain (38–40 kDa) and a basic β-chain (19–22 kDa), linked by disulfide bonds [13,23,26]. In contrast, vicilin is a trimeric protein, and the interactions between its subunits are not stabilized by disulfide bonds. Upon cleavage, several vicilin-derived subunits can appear, including Vαβγ (~50 kDa), Vαβ (~30–36 kDa), Vα (~20 kDa), Vβ (~13 kDa), and Vγ (~12 kDa) [27]. Another important vicilin-type protein is covicilin, which also has a trimeric structure and typically appears in SDS-PAGE gels at around 70 kDa [13,23,26].

Regarding the gel in native conditions, comparing the ratio 0:100 after the modification, clear changes in the band patterns can be observed. The bands related to legumin-αβ and vicilin-αβγ had a decrease in intensity. The molten state can promote the disruption of S-S bonds, leading to the cleavage of vicilin-αβγ and legumin-αβ, increasing the intensity of the subunits [28]. Jiang et al. [25], analyzing the effect of different pH levels in the pH-shifting, also observed the reduction in legumin-αβ intensity after pH-shifting at pH 12.

Comparing the native condition and reduced condition, a clear increase in the number of bonds in the gel can be observed. This increase is due to the use of β-mercaptoethanol, which acts as a reducing agent and promotes the cleavage of the disulfide bonds of the proteins [25]. One of the bands that is influenced by the use of β-mercaptoethanol is legumin-αβ, which disappeared, being converted into the bands referring to legumin-α and legumin-β. Other bands, such as vicilin-α, vicilin-β, and vicilin-γ, appear under reduced conditions due to the cleavage of S-S bonds by β-mercaptoethanol. Probably, its effect was more intense than the pH-shifting effect, causing a more significant effect in the S-S bonds.

### 3.4. Intrinsic Fluorescence

The intrinsic tryptophan (Trp) fluorescence is a sensitive method for detecting changes in protein tertiary structure, as it reflects alterations in the polarity of the Trp microenvironment. Therefore, it can provide strong indications of structural modifications induced by treatments such as pH-shifting [29].

**Figure 5:**
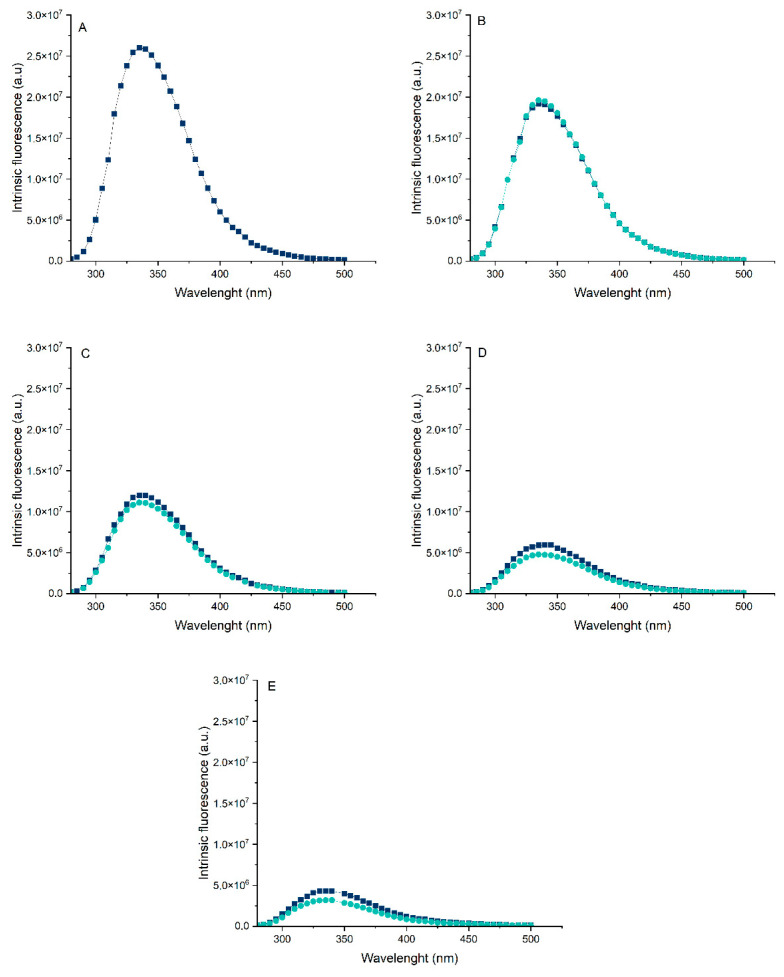
Intrinsic fluorescence of protein suspensions before and after pH-shifting: (**A**) 100:0 (**B**) 80:20 (**C**) 50:50 (**D**) 20:80 (**E**) 0:100 (casein/pea protein). Dark blue represents the suspensions before the modification, and light blue after the modification.

Before the pH-shifting treatment (Figure 5), a progressive decrease in intrinsic fluorescence intensity was observed as the proportion of pea protein increased. This variation among the samples can be attributed to the differing amounts of tryptophan residues in their structures. Casein contains approximately 1.55% more tryptophan than pea protein, which explains the higher fluorescence intensity observed in the 100:0 sample [14].

Regarding the pH-shifting treatment, no red or blue shift was observed in the emission spectra, with the maximum emission remaining at 335 nm. However, a decrease in fluorescence intensity was detected following the modification. In the 80:20 ratio, no significant structural changes were observed, likely because the amount of modified pea protein in the system was insufficient to alter the Trp microenvironment. In contrast, the 50:50 and 20:80 ratios showed a moderate reduction in fluorescence intensity, with the most pronounced decrease observed in the 0:100 sample. These results suggest that the pH-shifting treatment induced structural modifications in the proteins, altering the local environment around Trp residues.

Interestingly, a contrasting finding was reported by Zhang et al. [28], who observed an increase in intrinsic fluorescence after subjecting pea protein isolate to pH-shifting. However, in that study, the treatment time was relatively short (1 h), which may have limited the extent of Trp exposure. In the present study, the observed decrease in fluorescence intensity may be attributed to a quenching effect, potentially caused by increased solvent accessibility or interactions with quenching agents [14]. Despite the quenching, the reduction in fluorescence intensity still supports the hypothesis that the pH-shifting treatment promoted the exposure of Trp residues. This interpretation is further corroborated by the complementary results of solubility and zeta potential measurements presented earlier in this study.

### 3.5. Water-Holding Capacity

Water-holding capacity (WHC) refers to the ability of a protein matrix to retain water when subjected to centrifugal force. This property is crucial for determining the texture of food products such as yogurt and cheese [18].

**Figure 6:**
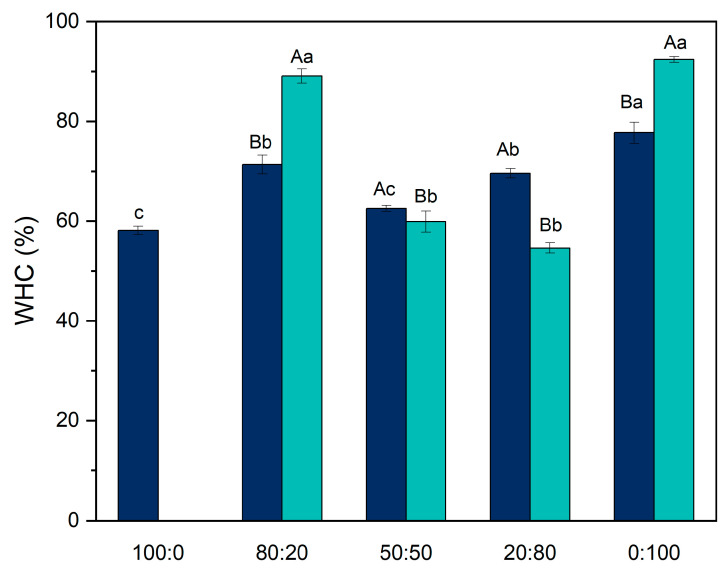
Water-holding capacity of hybrid gels formed by different ratios: 100:0. 80:20, 50:50, 20:80, and 0:100 (casein/pea protein) before and after pH-shifting. Dark blue represents the gel without modification, and light blue is the gel produced after pH-shifting. Uppercase letters mean differences between the treatments in the same ratios, and lowercase letters mean differences between the different ratios under the same treatments. Statistical differences were determined using one-way ANOVA followed by Tukey’s post hoc test (*p* < 0.05).

For the pure systems, before the pH-shifting treatment, the WHC values were 58.10 ± 0.86% for the 100:0 ratio and 77.70 ± 2.13% for the 0:100 ratio (Figure 6). Although these values are lower than those reported by Nascimento et al. [12], which were 82.6% and 98.3%, respectively, the observed trend remains consistent: casein gels retain less water than pea protein gels. According to the authors, this difference arises from the structural characteristics of the gels. Pea protein gels tend to form a denser, more interconnected network, allowing them to trap water more efficiently. In contrast, casein gels typically exhibit larger pores, reducing their water retention capacity.

In the mixed systems, no significant differences were observed between the 80:20 and 20:80 ratios. However, the 50:50 ratio exhibited the lowest WHC (62.54% ± 0.58%). In hybrid systems composed of both pea and casein proteins, the proteins may form separate networks rather than a cohesive matrix, resulting in a weaker gel structure and lower water retention [30,31,32].

After the pH-shifting treatment, the 0:100 ratio exhibited the highest WHC, reaching 92.39% ± 0.55%. Zhu et al. [16], investigating the effects of pH-shifting on pea protein gels, reported that the treatment led to the formation of more uniform pore sizes (3–5 μm). This more homogenous network structure allows the gel to retain water more effectively, as water molecules become more tightly trapped within the dense matrix.

Among the hybrid systems, the 80:20 ratio demonstrated the highest WHC (89.08% ± 1.43%) after treatment. In contrast, WHC decreased in the 50:50 and 20:80 ratios, dropping from 62.54% to 59.89% and from 69.58% to 54.62%, respectively. When higher amounts of pea protein are incorporated into a casein gel, the overall cohesion of the gel network may be compromised due to reduced structural and functional compatibility, which likely explains the reduced WHC [30]. However, in the 80:20 ratio, the structural modification of the pea protein likely enhanced its interaction with water, exerting a dominant effect on WHC.

### 3.6. Texture Profile Analysis (TPA)

During a TPA test, samples are subjected to two successive compressions, simulating the biting action of the human mouth. This process allows for the evaluation of the sample’s textural properties and behavior during mastication [31]. Hardness refers to the mechanical strength of a material and is calculated based on the maximum peak observed when a hard probe penetrates the gel [31,33].

As shown in Table 2, among the unmodified ratios, gel hardness decreases as more pea protein is incorporated into the system, dropping from 2.6735 ± 0.0224 N for the 100:0 ratio to 0.1452 ± 0.0100 N for the 0:100 ratio. This difference may be related to the water-holding capacity (WHC) of the gels. Casein gels have lower WHC, which allows for the formation of a more rigid structure. In contrast, pea protein has a higher WHC, which contributes to the reduction in hardness. Xia et al. [30], studying acid gels formed by pea proteins and casein micelles, also observed that the addition of pea protein to casein micelle gels led to a reduction in hardness.

Comparing the 80:20 and 50:50 ratios, after modification, the hardness decreased from 2.1129 N to 1.3828 N and from 0.8241 N to 0.4215 N, respectively. In the 80:20 ratio, the modified gel was able to retain more water in its structure, likely due to the unfolding of the pea protein, which contributed to the reduction in hardness. In the 50:50 ratio, despite structural alterations in the pea protein—evidenced by a decrease in particle size and an increase in zeta potential—the thermodynamic incompatibility between the proteins persisted. It is likely that after pH-shifting, pea proteins tended to self-associate even more, reducing the water-holding capacity (WHC) and, consequently, the gel hardness.

For the 20:80 ratio, after modification, hardness increased. This suggests that in mixed gels with a higher proportion of pea protein, the structural modification may enhance protein–protein interactions during gelation, overcoming the negative effect that casein may exert on the system and thereby increasing gel hardness. A similar trend was observed in the 0:100 ratio. Sun et al. [17], investigating the effect of pH-shifting on soy and potato proteins as well as their mixture, also reported an increase in gel hardness after pH-shifting, attributed to an increase in free sulfhydryl (SH) groups, which favors disulfide and covalent bond formation during gelation.

Chewiness is sometimes considered an undesirable attribute in foods such as meat, apples, and fish, but is regarded as essential in springy cheeses. It is closely correlated with gel hardness [31,34]. As with hardness, chewiness decreased as the proportion of pea protein increased. However, following pH-shifting, an increase in chewiness was observed.

Gumminess, defined as the energy required to disintegrate a semi-solid food in the mouth prior to swallowing [34], decreased with higher amounts of pea protein before modification. This reduction may be attributed to the structural characteristics of pea protein, which tends to form looser and more porous networks compared to casein, requiring less force to break down. After modification, gumminess increased for the 20:80 and 0:100 ratios—from 0.0369 N to 2.6644 N and from 0.0369 N to 0.2028 N, respectively—suggesting that the modification promotes pea protein–pea protein interactions, resulting in a firmer gel structure. In contrast, for the 80:20 and 50:50 ratios, the presence of pea protein in systems containing greater or equal amounts of casein appears to exert a disruptive effect on the gel matrix, reducing gumminess.

Springiness, which reflects how well gels return to their original height after compression [31], decreased with lower amounts of pea protein in the system, indicating that gels with higher casein content form more elastic and integrated structures [15]. When comparing samples before and after modification, springiness increased, suggesting that the modification may enhance gel elasticity. Gel resilience, also associated with elasticity, decreased as the proportion of pea protein increased, but rose after treatment—particularly in the 20:80 and 0:100 ratios—demonstrating the effectiveness of pH-shifting in promoting protein–protein interactions.

Cohesiveness reflects a gel’s resistance to structural breakdown and represents the amount of work required to disrupt internal bonds [34]. As the proportion of pea protein increased, cohesiveness decreased. A similar finding was reported by Xia et al. [30], who observed that gels with a 75:25 casein/pea protein ratio had a cohesiveness of approximately 0.7, which was halved at a 25:75 ratio. This reduction was attributed to a less homogeneous and consistent gel structure at higher pea protein levels. After pH-shifting, no significant differences in cohesiveness were observed, except for the 20:80 ratio, in which cohesiveness increased, likely due to enhanced protein–protein interactions.

## 4. Conclusions

This study demonstrates that pH-shifting is an effective tool for enhancing the techno-functional properties of hybrid systems composed of casein and pea proteins. The properties of these hybrid systems vary depending on the dominant protein in the suspension or gel, whether casein or pea protein predominates.

For the 80:20 ratio, the effect of pH-shifting was less pronounced. However, the presence of modified pea protein within the casein gel improved protein–protein interactions, leading to increased water-holding capacity (WHC) and reduced hardness. In the 50:50 and 20:80 ratios, despite the observed increase in solubility, the gels retained less water after gelation, accompanied by a decrease in gel hardness.

In the 0:100 ratio, the structural modifications induced by pH-shifting resulted in a significant increase in both solubility and WHC, producing a gel with distinct structural characteristics. These varying effects of pH-shifting on pea protein suggest that the structural and functional compatibility between the proteins plays a crucial role in determining the behavior of hybrid systems.

The results highlight the potential of pH-shifting in modifying pea protein and enhancing interactions between pea and casein in hybrid systems. The use of this technique is a promising approach in food formulation, especially for high-protein hybrid products aimed at balancing the functionality of casein with the sustainability of pea proteins. To the best of our knowledge, this is the first study to explore the impact of pH-shifting on hybrid casein–pea protein systems, providing valuable insights for future food product development.

## Figures and Tables

**Table 1:** Protein amount in the different ratios.

Ratio	Protein Amount in the Suspension
100:0	100% casein
80:20	80% casein and 20% pea protein
50:50	50% casein and 50% pea protein
20:80	20% casein and 80% pea protein
0:100	100% pea protein

**Table 2:** Texture profile analysis of hybrid gels for different protein ratios, 100:0, 80:20, 50:50, 20:80 and 0:100 (casein/pea protein), before and after pH-shifting.

Sample		Hardness (N)	Chewiness (N)	Resilience	Gumminess (N)	Springiness (mm)	Cohesiveness
Before pH-shifting	100:0	2.6735 ± 0.0224 ^a^	17.0898 ± 0.4235 ^a^	0.8947 ± 0.0103 ^a^	1.1628 ± 0.0503 ^a^	11.9540 ± 0.2416 ^a^	0.4342 ± 0.0288 ^abA^
	80:20	2.1129 ± 0.0455 ^bA^	13.0187 ± 2.0603 ^bB^	0.7998 ± 0.0165 ^abA^	0.8699 ± 0.1400 ^bA^	10.9078 ± 0.2395 ^aA^	0.4125 ± 0.0670 ^abA^
	50:50	0.8241 ± 0.0080 ^cA^	5.7489 ± 0.2305 ^cA^	0.7987 ± 0.0074 ^abA^	0.2983 ± 0.0733 ^cA^	7.8289 ± 0.5428 ^aA^	0.3778 ± 0.0964 ^aB^
	20:80	0.1116 ± 0.0104 ^dB^	0.4765 ± 0.1002 ^dB^	0.7516 ± 0.0387 ^bA^	0.0324 ± 0.0070 ^dB^	-	0.2857 ± 0.0800 ^bcA^
	0:100	0.1452 ± 0.0100 ^dB^	0.5379 ± 0.0531 ^dA^	0.6747 ± 0.0193 ^cA^	0.0369 ± 0.0040 ^dA^	-	0.2273 ± 0.0400 ^cA^
After pH-shifting	80:20	1.3828 ± 0.0571 ^aB^	8.6147 ± 0.7808 ^bA^	0.7573 ± 0.0217 ^aA^	0.5747 ± 0.0515 ^bA^	11.2789 ± 0.0283 ^aA^	0.4138 ± 0.0371 ^bA^
	50:50	0.4215 ± 0.0479 ^bB^	2.4497 ± 0.3426 ^bA^	0.8108 ± 0.0093 ^aA^	0.1651 ± 0.0217 ^bB^	7.9378 ± 0.6187 ^aA^	0.3933 ± 0.0601 ^bA^
	20:80	0.5267 ± 0.0135 ^bA^	39.8140 ± 3.0960 ^aA^	0.8431 ± 0.0330 ^aA^	2.6644 ± 0.1999 ^aA^	9.5650 ± 0.2996 ^a^	5.2000 ± 0.4228 ^aA^
	0:100	0.6179 ± 0.0694 ^bA^	3.0360 ± 0.6526 ^bA^	0.7849 ± 0.0318 ^aA^	0.2028 ± 0.0434 ^bA^	7.6442 ± 1.1931 ^a^	0.3333 ± 0.0882 ^bA^

Uppercase letters mean differences between the treatments in the same ratios, and lowercase letters mean differences between the different ratios under the same treatments. Statistical differences were determined using one-way ANOVA followed by Tukey’s post hoc test (*p* < 0.05).

## Data Availability

The original contributions presented in the study are included in the article, further inquiries can be directed to the corresponding author.
